# The correlation between serum levels of laminin, type IV collagen, type III procollagen N-terminal peptide and hyaluronic acid with the progression of post-COVID-19 pulmonary fibrosis

**DOI:** 10.3389/fcell.2024.1382244

**Published:** 2024-06-24

**Authors:** Dapeng Yu, Guangyue Yin, Jing Lei, Yijun Gong, Liang Zheng, Dahui He, Lihua Lei, Lei Sun

**Affiliations:** ^1^ School of Basic Medical Sciences, Guangdong Medical University, Dongguan, China; ^2^ Department of Clinical Laboratory, Hebei Petro China Central Hospital, Langfang, China; ^3^ School of Medical Technology, Guangdong Medical University, Dongguan, China; ^4^ Guangdong Provincial Engineering Technology Research Center for Autoimmune Laboratory Diagnostic Products, Shenzhen, China; ^5^ Department of Clinical Laboratory, Shenzhen Longgang District Third People’s Hospital, Shenzhen, China; ^6^ Department of Clinical Laboratory, Huaiji County Traditional Chinese Medicine Hospital, Zhaoqing, China; ^7^ Department of Clinical Laboratory, Changzhi People’s Hospital, Changzhi, China

**Keywords:** PASC, PF, PC19-PF, hyaluronic acid, laminin, procollagen III N-terminal peptide, type IV collagen, COVID-19

## Abstract

COVID-19 patients often suffer from post-COVID-19 acute sequelae (PASC). Pulmonary fibrosis has the most significant long-term impact on the respiratory health of patients, known as post-COVID-19 pulmonary fibrosis (PC19-PF). PC19-PF can be caused by acute respiratory distress syndrome (ARDS) or COVID-19-induced pneumonia. Individuals who experience COVID-19 pneumonia symptoms (including cough, shortness of breath, dyspnea on exertion, and desaturation) for at least 12 weeks after diagnosis, almost all develop PC19-PF. Extracellular matrix molecules: laminin (LN), type IV collagen (IV Col), procollagen III N-terminal peptide (PIIINP), and hyaluronic acid (HA) are involved in the development and progression of PC19-PF. This study aimed to investigate the relationship between the progression of PC19-PF and serum levels of laminin, IV COL, PIIINP, and hyaluronic acid. This retrospective study included 162 PC19-PF patients treated and 160 healthy controls who received treatment at Shenzhen Longgang District Third People’s Hospital, Hebei PetroChina Central Hospital and Changzhi People’s Hospital from January 2021 to December 2023. Serum levels of LN, IV COL, PIIINP, and HA were detected by chemiluminescence immunoassay using commercial kits. Predicted forced vital capacity percentage (FVC% pred), predicted carbon monoxide lung diffusion capacity percentage (D_L_CO% pred), high-resolution computed tomography (HRCT) scores were assessed, and patient mortality was compared with healthy controls. Serum levels of LN, IV Col, PIIINP, and HA were significantly higher in PC19-PF or CTD-ILD patients than in healthy controls (all *p* < 0.05), and they were further elevated in acute exacerbation cases (all *p* < 0.01). In patients, HA was positively associated with HRCT scores and negatively associated with FVC% pred and D_L_CO% pred (all *p* < 0.05). Serum levels of LN, IV COL, PIIINP, and HA were significantly lower in surviving patients than in those who deceased (all *p* > 0.05). Serum levels of LN, IV C, PIIINP, and HA may affect the progression of PC19-PF and may serve as indicators of PC19-PF severity.

## 1 Introduction

COVID-19 patients often suffer from post-COVID-19 acute sequelae (PASC). Pulmonary fibrosis (PF) has the most significant long-term impact on the respiratory health of patients, known as post-COVID-19 pulmonary fibrosis (PC19-PF). The term “post-COVID-19 pulmonary fibrosis” (PC19-PF) describes a pulmonary condition with yet to be established definitive prevalence, pathophysiology, and treatment strategies. Systematic review findings indicate a 7.0% prevalence of PC19-PF in five studies (Groff et al., 2021), contrasting with another report where 40% of COVID-19 patients developed acute respiratory distress syndrome (ARDS), 20% of which were severe cases (Wu et al., 2020). Although the exact prevalence of PC19-PF remains uncertain, early investigations of post-discharge patients reveal that over a third of COVID-19 survivors exhibit fibrotic lung changes (Liu et al., 2020).

PC19-PF is identified by persistent fibrotic changes on computed tomography (CT) scans during follow-up, linked to functional impairments (Tanin et al., 2021). The condition’s prevalence, pathogenesis, risk factors, and treatment options require further elucidation. Patient classification post-COVID-19 includes: (1) ongoing symptoms post-acute phase, (2) deteriorated quality of life and functional status *versus* pre-COVID-19, and (3) sustained or worsening radiological and pulmonary anomalies (Oronsky et al., 2021).

Many individuals recovering from COVID-19 experience lingering symptoms, with growing evidence of subacute and long-term sequelae. PC19-PF emerges as a critical long-term complication, notably affecting patient care and health outcomes (Mohammadi et al., 2022). ARDS and pneumonia during the acute phase of COVID-19 can lead to PC19-PF, characterized by alveolar inflammation, scarring in lung tissue, epithelial dysfunction, continuous fibroblast proliferation and excess extracellular matrix (ECM) deposition (Loomis-King et al., 2013).

Three years post-pandemic, numerous COVID-19 survivors show signs of fibrotic lung changes and altered pulmonary function, suggesting a potential association with restrictive lung disease, a key characteristic of PC19-PF. Hence, PC19-PF is a significant complication during the latter stages of COVID-19 illness, similar to SARS (Severe Acute Respiratory Syndrome) and MERS (Middle East Respiratory Syndrome), with ARDS (Acute Respiratory Distress Syndrome) being a prevalent critical complication in SARS-CoV-2 infections (Mohammadi et al., 2022). Nonetheless, the exact pathology of PC19-PF and acute COVID-19 remains ambiguous (Mylvaganam et al., 2021). Pulmonary fibrosis could result from inadequate lung injury resolution or excessive healing, potentially exacerbated by cytokines from pulmonary epithelial cells and macrophages, as well as detrimental mechanical ventilation effects (Pelosi and Rocco, 2008).

PC19-PF progression often leads to adverse outcomes, including pulmonary hypertension, heart complications, and a limited survival prognosis of 3–5 years (Loomis-King et al., 2013). COVID-19’s entry through ACE-2 receptors affects type II alveolar cells, leading to diffuse alveolar damage (DAD) and an imbalance in collagen homeostasis, fostering ECM accumulation (Virk and Fraire, 2015). Previous studies have also demonstrated that four ECM molecules are involved in the development of PC19-PF (Oronsky et al., 2021; Solomon et al., 2021; Torres-Castro et al., 2021; Mohammadi et al., 2022).

In the past, the serum levels of four ECM molecules have been tested as biological markers for assessing liver fibrosis and predicting the prognosis of patients with liver diseases (El-Mezayen et al., 2015; Babic et al., 2003; Neuman et al., 2016). Additionally, patients with pulmonary function deterioration and radiographic progression exhibited higher PIIINP and HA levels than those with stable disease (Bjermer et al., 1989). Research on bleomycin-induced pulmonary fibrosis in mice corroborates that increased collagen I deposition, a phenomenon observed alongside other ECM components like laminin and type IV collagen, can be indicative of pulmonary fibrosis development (Teles-Grilo et al., 2005; Lee et al., 2014; Morales-Nebreda et al., 2015). The serum levels of certain ECM molecules, such as the III procollagen N-terminal peptide (PIIINP) and hyaluronic acid (HA), are also considered indicative of fibrosis progression in PC19-PF (Kivirikko and Myllylä, 1985; Low et al., 1992; Lennon and Singleton, 2011; Inokoshi et al., 2013).

This study draws parallels from liver fibrosis researches, where ECM serum levels, alongside liver biochemistry markers, have facilitated pulmonary fibrosis assessment and prognosis (El-Mezayen et al., 2015; Babic et al., 2003; Neuman et al., 2016). By analyzing serum levels of LN, IV COL, PIIINP, and HA in PC19-PF patients against pulmonary function tests and high-resolution computed tomography (HRCT) scores, this research seeks to elucidate their relationship with disease severity and survival prognosis in PC19-PF.

## 2 Materials and methods

### 2.1 Research design

This retrospective study included a total of 162 patients with PC19-PF who received treatment at Shenzhen Longgang District Third People’s Hospital, Hebei PetroChina Central Hospital and Changzhi People’s Hospital from January 2021 to December 2023. All experimental procedures are in comply with ethical guidelines and regulations. All data are anonymous. Written informed consent documents were obtained from each participant.

### 2.2 Study participants

The inclusion criteria for patients: Patients with confirmed COVID-19: Based on Diagnosis and treatment guideline for COVID-19 (Trial Version 9) in China. Patients diagnosed with PC19-PF: Based on available medical imaging, serum LN, IV COL, PIIINP and HA detection results, as well as clinical diagnosis.

The revised diagnosis criteria of PC19-PF acute exacerbation (AE-PC19-PF): Persistent worsening of shortness of breath within 30 days, Pa_O2_/Fi_O2_ ratio of less than 225, a decrease over time in Pa_O2_ of 10 mmHg or greater, and increased shadowing on chest imaging. (Harold et al., 2007). The COVID-19 diagnosis history was based on patients’ self-report thus the actual infection date was not available for the current study. The biomarker sampling was performed in hospitalized patients during the admission check-up, and in volunteers during their routine physical examination post. Therefor the participants are categorized by the symptoms not the post infection duration. The lung function test and the biomarker test were tested the same time during the check-ups.

A total of 160 healthy individuals of matching age who had routine physical examination at the above hospitals were included as controls. In December 2022, the Chinese government adjusted the COVID-19 prevention and control strategy, restarting social and economic activities. According to different epidemiological data, the cumulative infection rate of urban population in China is no less than 95.46% (Halllgren et al., 1989). Most of these infected individuals developed mild symptoms and recovered quickly due to COVID-19 vaccination program and less virulent virus strains after multiple mutation from 2020–2022. Some recovered healthy patients are concerned about the impact of long-term COVID-19, and they requested cardiac or pulmonary function tests during physical examinations from hospitals. All participants who were categorized as healthy controls had a history of COVID-19 infection and recovery.

### 2.3 Measurement of serum levels of laminin, type IV collagen, type III procollagen N-terminal peptide, and hyaluronic acid

Venous blood (2 mL) was collected from each participant after overnight fasting. Blood samples were centrifuged at 3,000 *g* at 4°C for 5 min, and the supernatant was kept in a cryopreservation tube at −20°C for testing. The levels of LN, IV COL, PIIINP and HA in the supernatant were measured by chemiluminescence immunoassay analyzer iFlash 3,000 with commercial kits (Shenzhen YHLO Biotech Co., Ltd., Shenzhen, China) in accordance with the manufacturer’s instructions.

#### 2.3.1 Principles of LN, IV COL, PIIINP and HA assays

##### 2.3.1.1 HA: The iFlash-HA assay is a competitive immunoassay using direct chemiluminometric technique

In the first step, samples, HA-coated paramagnetic microparticles, and HA-binding protein (HABP) are added. HA in the sample and HA-coated paramagnetic microparticles bind competitively to the HABP to form a HA-HABP complex. This complex then binds to added acridinium-ester-labeled anti-HABP antibodies, resulting in the formation of an immune complex.

In the second step (washing), under the magnetic field, magnetic particles are adsorbed to the wall of the reaction tube, and the unbound materials are washed away by the wash buffer.

In the third step (signal triggering and measuring): The pre-trigger and trigger solutions are added to the reaction mixture. The resulting chemiluminescent reaction is measured as relative light units (RLUs). An inverse relationship exists between the amount of HA in the sample and the RLUs detected by the iFlash optical system. Results are determined via a calibration curve, which is generated by four-point calibration and a master curve provided from the reagent QR code.

##### 2.3.1.2 LN: The iFlash-Laminin assay is a sandwich immunoassay

In the first step (incubation), sample, paramagnetic particle coated Laminin antibody and acridinium-ester-labeled Laminin antibody react to form an immune complex. The second, third step are similar to HA, except it is three-point calibration for LN instead of four-point calibration in HA.

##### 2.3.1.3 IV COL: The iFlash-CoI Ⅳ assay is a sandwich immunoassay

In the first step (incubation), CoI Ⅳ in the sample, paramagnetic particles coated CoI Ⅳ antibody and acridinium-ester-labeled CoI Ⅳ antibody react to form an immune complex. The second, third step are similar to HA, except it is three-point calibration for IV COL instead of four-point calibration in HA.

##### 2.3.1.4 PIIINP: The iFlash-PⅢPN-P assay is a sandwich immunoassay

In the first step (incubation), PⅢPN-P in the sample, anti-PⅢPN-P coated paramagnetic particles and anti-PⅢPN-P acridinium-ester-labeled conjugate react to form an immune complex. The second, third step are similar to HA, except it is three-point calibration for PIIIPN-P instead of four-point calibration in HA.

### 2.4 Scoring of chest high-resolution computed tomography (HRCT)

Background: Among COVID-19 patients from Wuhan, China, more than half of them had abnormal CT scan results 6 month after first admission, and the most common changes in imaging results are pulmonary interstitial tissue with ground glass opacity. CT manifestations of PASC (post-acute sequelae of coronavirus disease 2019) should be standardized as being dominated by ground glass opacity, fibrosis, and a mixed pattern of ground glass opacity and fibrosis (Solomon et al., 2021; Torres-Castro et al., 2021). The British Thoracic Society had a recommendation that all critically ill COVID-19 survivors should undergo pulmonary function test (PFT) every 3 months after their discharge from hospital, or, if there are abnormalities on medical imaging, even patients with mild to moderate symptoms should also perform PFT (George et al., 2020). High resolution CT (HRCT) of the chest is currently considered the “gold standard” for diagnosing pulmonary fibrosis in clinical practice, and it is widely accepted to use the Warrick scoring system (Warrick et al., 1991) to score HRCT imaging findings of patients. Including severity and lesion range scores:

1 point for ground glass shadow, two points for irregular pleural edge, three points for thickening of interlobular septum or subpleural line sign, four points for honeycomb shadow, and five points for subpleural air cyst; Involving one to three lung segments for one point, four to nine lung segments for two points,>9 lung segments for three points, with a maximum of 30 points. A Warrick score of ≥1 is defined as positive. Semi quantitative evaluation of different degrees of pulmonary fibrosis: mild (<8 points), moderate (8–15 points), severe (>15 points). Table 1 is the HRCT scoring criteria.

**TABLE 1:** HRCT scoring criteria.

Lesions and lung segments	Score
Parenchymal abnormalities	Severity score
Ground glass opacities	1
Irregularities in the pleural margins	2
Septal/subpleural lines	3
Honeycombing	4
Subpleural cysts	5
Number of lung segments	Extent score
1–3	1
4–9	2
>9	3

HRCT, score = severity score + extent score (0–30).

### 2.5 Pulmonary function assessment

The pulmonary function of PC19-PF patients was evaluated using the percentage of predicted forced vital capacity (FVC% pred) and the percentage of predicted lung diffusion capacity for carbon monoxide (D_L_CO% pred). FVC% pred is an indicator for pulmonary ventilation capacity, and D_L_CO% pred is an indicator for pulmonary diffusion capacity. They are considered as important markers for assessing the pulmonary function of PC19-PF patients (King et al., 2014).

### 2.6 Follow-up

Follow-up for the 162 PC19-PF patients were carried out by outpatient department or via telephone interviews in the above-mentioned hospitals. The follow-up ended on 31 December 2023. The total mortality rate and 1-year mortality rate were recorded. Serum levels of LN, IV COL, PIIINP, and HA were compared between deceased and surviving patients.

### 2.7 Statistical analysis

Statistical analysis was performed using software SPSS 16.0 (Chicago, Illinois). Continuous variables were expressed as mean ± standard deviation. F-test in a one-way analysis of variance (ANOVA) followed by Tukey’s test was used to analyze the differences between different groups, and *p* < 0.05 was considered as statistically significant. Two-factor linear regression analysis was conducted to analyze the relationships between serum levels of LN, IV COL, PIIINP, HA and FVC% pred, D_L_CO% pred, HRCT scores.

## 3 Results

### 3.1 Clinical characteristics

Table 2 is the flowchart of the patient screening (Table 2). A total of 162 patients with PC19-PF who received treatments in the above-mentioned hospitals from January 2021 to December 2023 were screened. The whole study included a total of 162 patients, and 160 healthy individuals with similar ages as the control group. The proportion of smokers in the PC19-PF group (84.0%) was higher than that in the control group (37.5%). Among the 162 patients with PC19-PF, 39 cases were diagnosed with AE-PC19-PF, and the remaining 123 patients had stable PC19-PF.

**TABLE 2:** Demographics and clinical characteristics of study participants.

	PC19-PF (n = 162)	Control (n = 160)
Man/woman (ratio)	110/52 (2.12:1)	79/81 (1:1.03)
Age (mean ± SD), y	65 ± 4	62 ± 3
Smoker, n (%)	136 (84.0)∗	60 (37.5)
*FVC, L (n = 258)	2.5 ± 0.9 (n = 123)	2.7 ± 0.54
*FVC, % pred (n = 258)	69.8 ± 17.6 (n = 123)	96 ± 15
*D_L_CO, % pred (n = 258)	52.7 ± 22.7 (n = 123)	96 ± 15
HRCT score	17.3 ± 7.5	NA
*AE	39	0

Control: healthy individuals. AE, acute exacerbation D_L_CO, carbon monoxide diffusion capacity; FVC, forced vital capacity, PC19-PF, post-COVID-19, Pulmonary Fibrosis; NA = not applicable; SD, standard deviation.

*Patients with AE-Post-COVID-19, Pulmonary Fibrosis (n = 39) were not able to undergo pulmonary function test due to their severe situations.

### 3.2 Serum levels of LN, IV COL, PIIINP and HA, pulmonary function, and HRCT scores

**FIGURE 1:**
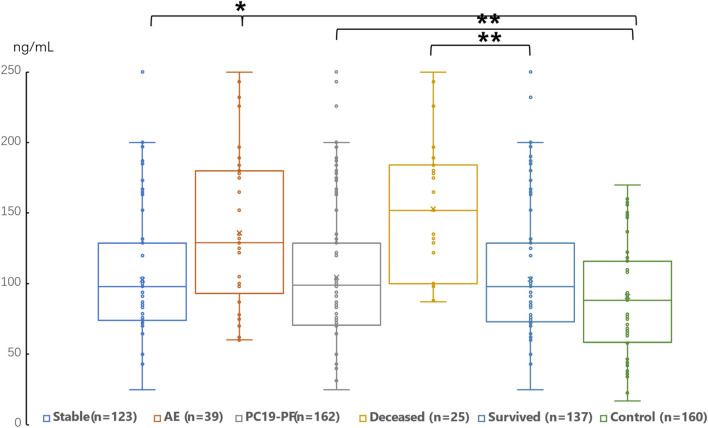
Comparison of the serum level of LN in AE patients, stable patients, deceased patients within 1 year, surviving patients, sum PC19-PF patients, and control.

**FIGURE 2:**
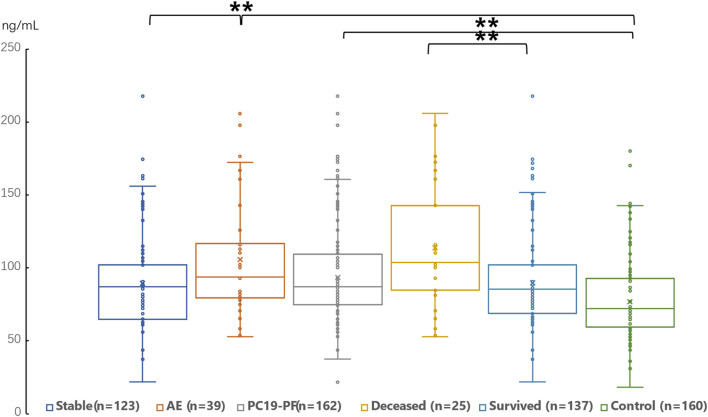
Comparison of the serum level of IV Col in AE patients, stable patients, deceased patients within 1 year, surviving patients, sum PC19-PF patients, and control.

**FIGURE 3:**
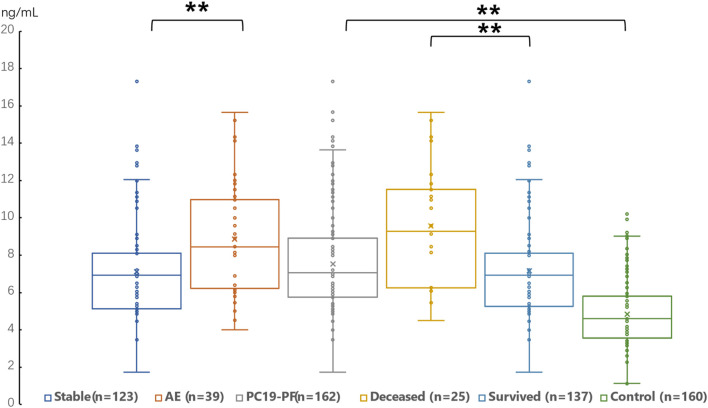
Comparison of the serum level of PIIINP in AE patients, stable patients, deceased patients within 1 year, surviving patients, sum PC19-PF patients, and control.

**FIGURE 4:**
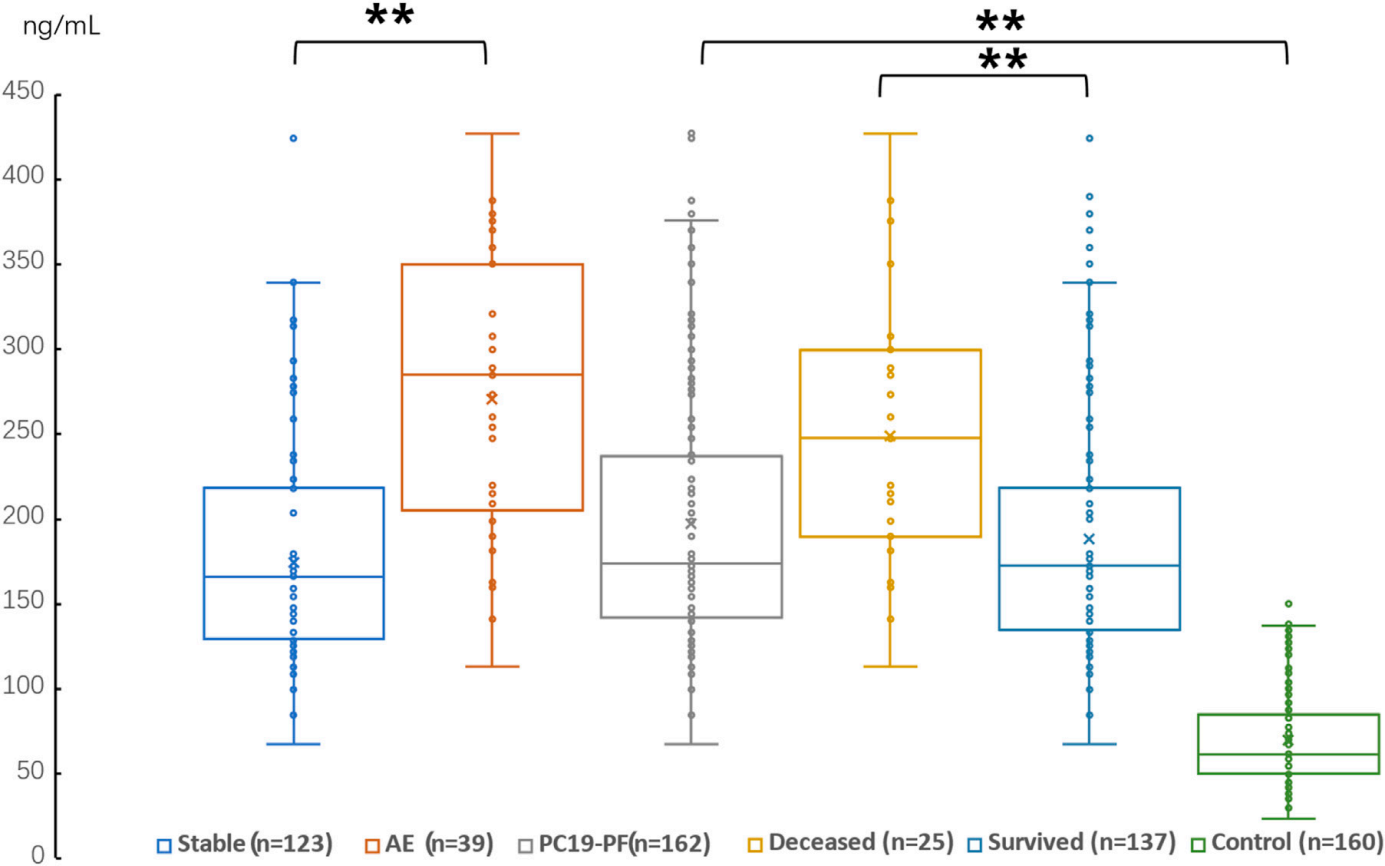
Comparison of the serum level of HA in AE patients, stable patients, deceased patients within 1 year, surviving patients, sum PC19-PF patients, and control.

**FIGURE 5:**
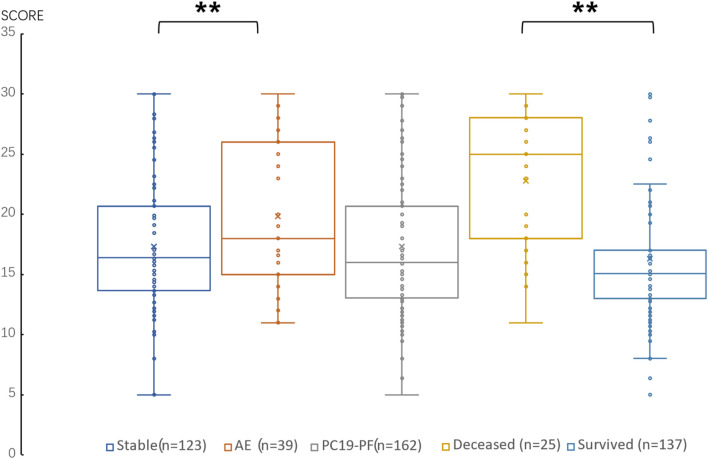
Comparison of the score of HRCT in AE patients, stable patients, deceased patients within 1 year, surviving patients and sum PC19-PF patients.

The serum levels of LN, IV COL, PIIINP and HA were significantly higher in the PC19-PF group than in the control group (all *p* < 0.05, Table 3; Figures 1–4). Notably, the serum levels of PIIINP and HA in the PC19-PF patients were 1.6 times higher than those in the control group (Table 3; Figures 3, 4), and the serum levels of LN and IV COL were approximately 20% higher in the PC19-PF group than in the control group (Table 3; Figures 1, 2). Serum levels of LN, IV COL, PIIINP, and HA had significantly negative correlation with FVC (%pred) and D_L_CO (%pred) in PC19-PF patients (all *p* < 0.01, Table 4), and they were significantly positively correlated with HRCT scores (*p* < 0.01, Table 4; Figure 5).

**TABLE 3:** Comparison of the levels of LN, IV COL, PIIINP, and clinical characteristics.

	PC19-PF					Control (n = 160)	*F*	*p*
	Stable (n = 123)	AE (n = 39)	Deceased (n = 25)	Survived (n = 137)	Sum (*n* = 162)			
LN, ng/mL	102.74 ± 43.43	135.86 ± 52.83	152.98 ± 50.47	103.00 ± 43.27	110.10 ± 47.19	91.04 ± 48.60	4.21	0.016*
IV Col, ng/mL	89.54 ± 32.73	105.82 ± 39.99	113.73 ± 45.12	89.76 ± 31.89	94.07 ± 36.52	76.68 ± 31.82	13.954	0.000**
PIIINP, ng/mL	7.11 ± 1.95	8.86 ± 2.99	9.58 ± 3.50	7.16 ± 1.88	7.53 ± 2.89	4.85 ± 0.89	60.392	0.000**
HA, ng/mL	174.24 ± 47.54	270.68 ± 92.27	249.12 ± 85.62	188.03 ± 67.51	197.27 ± 73.66	70.01 ± 34.60	289.799	0.000**
HRCT score	16.5 ± 7.3	19.8 ± 7.8	22.8 ± 7.8	16.3 ± 7.2	17.3 ± 7.5	NA	7.993	0.005**

*p < 0.05 **p < 0.01.

PC19-PF, post-COVID-19, Pulmonary Fibrosis; HA = hyaluronic acid; IV, Col = type IV, collagen; LN, laminin; PIIINP, type III, procollagen N-terminal peptide.

**TABLE 4:** Correlation of serum levels of LN, IV COL, PIIINP and HA to percentage of forced vital capacity in the prediction value or FVC (%pred), percentage of diffusing capacity of the lung for carbon monoxide in the prediction value or D_L_CO (%pred), and HRCT score.

	LN		IV Col		PIII NP		HA	
PC19-PF	*r*	*p*	*r*	*p*	*r*	*p*	*r*	*p*
FVC(%pred)	0.391	<0.01	0.590	<0.01	0.536	<0.01	0.468	<0.01
D_L_CO(%pred)	0.232	<0.01	0.603	<0.01	0.370	<0.01	0.196	0.03
HRCT score	0.479	<0.01	0.535	<0.01	0.406	<0.01	0.666	<0.01

D_L_CO, carbon monoxide diffusion capacity; FVC, forced vital capacity; HA = hyaluronic acid; IPF, idiopathic pulmonary fibrosis; IVC, type IV, collagen; LN, laminin; PIIINP, type III, procollagen N-terminal peptide, *r* = correlation coefficient.

The serum levels of LN, IVC, PIIINP, and HA showed significant differences in AE, patients, stable patients, deceased patients within 1 year, surviving patients, sum PC19-PF, patients, and control with statistical significance.

The AE, had significantly higher serum levels of LN, IVC, PIIINP, and HA than patients with stable PC19-PF.

All PC19-PF, patients including AE, cases had significantly higher serum levels of LN, IVC, PIIINP, and HA than control.

The deceased patients had significantly higher serum levels of LN, IV, col; PIIINP, and HA than those of surviving patients.

### 3.3 Correlation of serum levels of LN, IV COL, PIIINP, and HA with survival prognosis

162 patients of PC19-PF were followed up between 4 and 156 weeks, with a median follow-up time of 80 weeks. The 1-year mortality rate was 15.4% (25/162), and the overall mortality rate in the PC19-PF group was 33.95% (55/162). Serum levels of LN, IV COL, PIIINP, and HA were significantly higher in deceased patients within 1 year than surviving patients (*p* < 0.01, Table 3), indicating that the serum levels of these four ECM molecules may be associated with survival prognosis.

## 4 Discussion

Four years into the COVID-19 pandemic, there is mounting evidence demonstrating that many COVID-19 patients develop fibrotic sequelae and changes in lung function in post-COVID stage. It suggests that restrictive lung disease is a hallmark PC19-PF patients. Thus, PF has been considered as one of the complications of severe COVID-19 infection seen in the third stage of COVID-19 patients. Whether post-COVID-19 fibrotic lung alterations are stable or progressing, like in fibrotic lung illnesses such idiopathic pulmonary fibrosis (IPF), it is important to ascertain for patient management and treatment. Different challenges in the diagnosis of PC19-PF must be thoroughly investigated as soon as possible. So, finding biomarkers related to post COVID-19 pulmonary fibrosis is particularly important.

Up to date, there have been a collection of studies attempting to establish a correlation between pulmonary fibrosis with damage and alveolar epithelial cells, as well as fibroproliferation, and matrix remodeling biomarkers such as LN, IV COL, PIIINP, and HA. The abnormal distribution of the α3 LN subunit was observed in the basement membranes of fibrotic areas in the pulmonary fibrosis mice (Luisa et al.). Elevated levels of collagen I and IV expression were found deposited around newly formed pulmonary blood vessels in the lung of a bleomycin-induced pulmonary fibrosis rat model (Teles-Grilo et al., 2005). Similarly in humans, increased levels of XII, XIV, Type I collagens (Tzortzaki et al., 2006), PIIINP (Bjermer et al., 1989; Low et al., 1992) and HA (Bjermer et al., 1989; Inokoshi et al., 2013) were found in PF patients when compared to healthy individuals. It is worth noting that elevated PIIINP and HA levels were reported to associate with more severe disease progression (Milman et al., 1995), but serum PIIINP levels were negatively correlated with total lung capacity in patients with pulmonary fibrosis (Heikkila et al., 2013). However, these studies had limitations, either only based on a small number of patients, or one or two ECM molecules, or inadequate observation time (Bjermer et al., 1989; Low et al., 1992; Milman et al., 1995; Inokoshi et al., 2013; Virk and Fraire, 2015). After the COVID-19 pandemic, there have been a considerable number of long-term complications of COVID-19 and long COVID-19 in the world (Groff et al., 2021). The purpose of this study is to assess the clinical diagnostic value of four ECM molecules in PC19-PF patients.

The current study followed up a much larger cohort of 162 patients for up to 156 weeks, and found that the elevated serum levels of four ECM molecules were significantly associated with increased mortality (*p* < 0.01). To our knowledge, this is the first study to demonstrate a strong correlation between the four ECM biomarkers and PC19-PF progression, lung function parameters, and HRCT scores. Additionally, these biomarkers are significantly negatively correlated with FVC% pred and D_L_CO% pred.

AE-IPF is a severe disease that is often associated with high mortality and poor patient prognosis (Harold et al., 2007). It has been reported that the mortality rate associated with AE-IPF ranges from 71% to 78% (Kubo et al., 2005; Kim et al., 2006), and the incidence of AE-IPF in IPF patients is 35%–57% (Kubo et al., 2005; Kim et al., 2006; Song et al., 2011). However, there is a lack of knowledge of the incidence of AE-PC19-PF. In the current study, the incidence of AE-PC19-PF was 24.1%, and the mortality rate of AE-PC19-PF was 64.1%, which was slightly lower than previous IPF reports. Combining the finding of the serum level difference of the four ECM molecules in deceased patients and the survivors, it indicates the biomarkers as the survival prognosis for patients with AE-PC19-PF.

In the lungs of ARDS patients, the proliferation and activation of fibroblasts after lung injury led to an increase in ECM molecules secretion, resulting in the accumulation of a large amount of ECM molecules (Hu et al., 2016; Xu et al., 2020), which play important roles in tissue development, remodeling, and repair (Petrey and de la Motte, 2014), as well as inflammation, immune response, and tissue damage (Liang et al., 2016). It is reported that in patients with COVID-19, especially severe patients, excessive collagen deposition can lead to pulmonary fibrosis, pulmonary ventilation and gas exchange dysfunction, respiratory failure and irreversible damage (Tran et al., 2022; Patrucco et al., 2023). This study aligns well with the above reports and further reinforces the link to disease progression by demonstrating differential ECM levels in critically ill patients and mild patients.

Besides respiratory system injury, cardiac complications such as myocardial injury and heart failure are also common in patients with COVID-19 infection (Brogi et al., 2022; Xie et al., 2022; Ntchana et al., 2023). It is conceivable that since ECM molecules are important components of myocardial cells and myocardial interstitium for maintaining heart structure, facilitating force transmission between myocardium, and ensuring myocardial contractility (Dupuy et al., 2019), increased levels of ECM molecules are associated with the progression of myocardial fibrosis, heart failure, and acute myocardial infarction (Wellens, 2019). Taken together, our findings provide insights into possible biomarkers for the detailed pathological process of COVID-19. Thus, we propose that the elevation of serum ECM levels may serve as an early diagnostic indicator for cardiac complications in COVID-19 patients.

The current study revealed a positive association between the severity of PC19-PF and the serum levels of LN, IN-COL, PIIINP, and HA. While the findings are significant, we feel the need for further investigation to reveal the role of these four ECM factors in the progression of PC19-PF, which was not possible during the investigation. Future studies will include longitudinal assessments of the four ECM factors within a PC19-PF patient, where the levels of these ECM factors are monitored through serial measurements.

Conclusion: Significantly higher serum levels of LN, IVC, PIIINP, and HA are found in patients with PC19-PF when compared with healthy individuals, and in patients with AE when compared with patients with stable disease. HRCT scores positively correlated with serum levels of the 4 ECM molecules, whereas FVC% pred and D_L_CO %pred negatively correlated with them. Surviving patients had significantly lower serum levels of the 4 ECM molecules than deceased patients. These results suggest that the panel of LN, IVC, PIIINP, and HA may serve as the biomarkers to reflect PC19-PF progression.

## Data Availability

The original contributions presented in the study are included in the article/Supplementary material, further inquiries can be directed to the corresponding authors.

## Abbreviations

AE = acute exacerbation, D_L_CO = carbon monoxide diffusion capacity, ECM = extracellular matrix, FVC = forced vital capacity, HA = hyaluronic acid, HRCT = high resolution computed tomography, ILD = interstitial lung disease, IPF = idiopathic pulmonary fibrosis, IV Col = type IV collagen, LN = laminin, PIIINP = type III procollagen N-terminal peptide, PASC = post-acute sequelae of coronavirus disease 2019, PF = Pulmonary fibrosis (PF), PC19-PF = post-COVID-19 pulmonary fibrosis.
